# Language-Based Inequities in Transfusion Practices with Obstetric Hemorrhage

**DOI:** 10.1007/s10995-025-04118-2

**Published:** 2025-07-08

**Authors:** Alexa Cohen, Samantha Goulding, Carly Pickett, Beatrice Lynch, Osaro Obanor, Melissa Peskin-Stolze, Pe’er Dar, Georgios Doulaveris

**Affiliations:** Division of Fetal Medicine and Ultrasound, Department of Obstetrics and Gynecology and Women’s Health, Montefiore Medical Center/Albert Einstein College of Medicine, Bronx, USA

**Keywords:** Disparities, Health inequities, Language, English-speaking, Postpartum hemorrhage, Transfusion, Maternal morbidity

## Abstract

**Objectives:**

Inequities in race, ethnicity and socioeconomic status have been well documented in postpartum hemorrhage (PPH) and hemorrhage-associated morbidity. However, little is known about the impact of language barriers on maternal outcomes in PPH. Our study aim was to investigate language-based inequities in maternal outcomes among gravidas with PPH.

**Methods:**

This is a retrospective cohort of patients with PPH who delivered at an urban academic institution between January 2020 and December 2022. Maternal language is categorized as English primary language (EPL) or non-English primary language (NEPL). PPH is defined as a quantitative blood loss (QBL) greater than 1000 mL within 24 h of delivery. QBL is a calculated measurement of peripartum and postpartum blood loss. Primary outcome is transfusion of packed red blood cells (pRBC). Secondary outcomes include transfusion of 4 + units of pRBC, disseminated intravascular coagulation (DIC) and admission to intensive care unit (ICU). Multivariable logistic regression was used to estimate the association of primary language with maternal outcomes.

**Results:**

1723 patients with PPH were included: 1314 (76.3%) with EPL and 409 (23.7%) with NEPL. English-speaking and non-English speaking patients had similar QBL rates (1530.2 ± 634.2 vs 1496.0 ± 668.1, p = 0.3). However, transfusion rates were lower in those with NEPL, when compared to EPL (28.2% vs 22.9%, p = 0.039). After adjusting for age, race/ethnicity, nulliparity, body mass index, pre-eclampsia and pre-delivery anemia, gravidas with NEPL were less likely to be transfused compared with EPL (aOR 0.7, 95% CI 1.012–1.806, p = 0.04). Rates of DIC, ICU admission and transfusion of 4 + units of pRBC were similar between groups.

**Conclusions for Practice:**

Despite a similar postpartum blood loss, patients with NEPL had lower rates of blood transfusion in PPH compared to patients with EPL. Further research is needed in health literacy and language proficiency that may impede access to transfusion in patients with PPH.

## Significance

In our study examining language-based inequities in maternal morbidity, we found that amongst patients with obstetric hemorrhage, non-English speaking patients were less likely to receive a blood transfusion compared to English speaking patients, despite a similar blood loss.

## Introduction

Postpartum hemorrhage (PPH) is a leading preventable cause of maternal morbidity and mortality, accounting for 11% of maternal deaths in the United States (Bienstock et al., 2021; Corbetta-Rastelli et al., 2022; James et al., 2022; Postpartum Hemorrhage, 2017; Quantitative Blood Loss in Obstetric Hemorrhage, 2019). From 2000 to 2019, the rates of PPH have steadily increased from 2.7% to 4.3% (Corbetta-Rastelli et al., 2022). PPH may lead to catastrophic sequalae including transfusion, disseminated intravascular coagulation (DIC), admission to the intensive care unit (ICU), and hysterectomy (Corbetta-Rastelli et al., 2022; Quantitative Blood Loss in Obstetric Hemorrhage, 2019).

Racial and/or ethnic inequities in obstetric hemorrhage and hemorrhage-related morbidity have been well substantiated in the literature (Collier & Molina, 2019; Guan et al., 2023; Gyamfi-Bannerman et al., 2018; James et al., 2022; Okunlola et al., 2022; Patek & Friedman, 2023; Postpartum Hemorrhage, 2017; Postpartum Hemorrhage, 2017; Sabato et al., 2020). Effective cross-cultural and language communication is important in eliminating potential inequities in health care. To date, there is limited available data on the impact of a primary language barrier on maternal outcomes in patients whose birth is complicated by PPH. Within obstetrics, language disparities have been demonstrated in obstetric birth trauma among non-English speaking patients in Hawaii, access and utilization of prenatal care amongst language-discordant migrant patients in France, and unscheduled cesarean delivery in non-English speaking patients in New York City (Comfort et al., 2023; Eslier et al., 2023; Sentell et al., 2015).

In 2022, 30.7% of households in New York City reported a non-English speaking household (Dietrich et al., 2022). In our study, we sought to examine the association between primary language and maternal outcomes with obstetric hemorrhage in a racially and ethnically diverse patient population in New York City. We hypothesized that non-English-speaking patients whose birth is complicated by obstetric hemorrhage will be less likely to receive a blood transfusion.

## Materials and Methods

This was a retrospective observational cohort study of patients who had a vaginal or cesarean delivery complicated by PPH at a single urban academic institution between January 1, 2020 and December 31, 2022. Study approval was obtained by our institutional review board, and a waiver of consent was granted given the retrospective nature of the study. Cases were identified by querying the electronic medical record (EMR) for quantitative blood loss (QBL) greater than 1000 mL within 24 h of delivery; this was then cross-referenced with the clinical EMR to confirm the diagnosis. Individuals with placenta accreta spectrum, placenta previa or who did not deliver at our institution were excluded from the study.

PPH was defined as a QBL of more than 1000 ml of blood within the first 24 h after delivery. For improved accuracy, quantitative measurement of blood loss was used instead of visual estimation (Quantitative Blood Loss in Obstetric Hemorrhage, 2019). At our institution, blood loss quantification starts after birth and consists of a calculation that incorporates a combination of pooled blood in the under-buttocks drape, and weighted blood-soaked towels and laparotomy sponges.

Patient demographic, maternal and obstetric characteristics were extracted from the EMR via individual chart review and transcribed in a deidentified manner by members of the research team. Data was reviewed for accuracy and completeness by research team leaders and then stored in a password-protected database. Only pertinent members of the team had access to this database.

The primary exposure variable was maternal primary language, which was categorized as English primary language (EPL) and non-English primary language (NEPL). NEPL included Spanish, French, Bengali, Arabic or other languages. Maternal primary language was self-reported. The primary outcome measure examined was transfusion of packed red blood cells (pRBCS). Currently there is no formal guidance on recommended threshold for postpartum transfusion. At our institution, transfusion of pRBCs is typically recommended for hemoglobin < 7 g/dl or > 7 g/dl with anemia symptoms. Secondary outcomes included transfusion of 4 or more units of pRBCs, disseminated intravascular coagulation (DIC) and admission to the intensive care unit (ICU).

Power analysis was conducted to achieve an 80% power and two-sided α value of 0.05. Based on United States population data, rate of blood transfusion in PPH was 12.6% (Corbetta-Rastelli et al., 2022); therefore, we projected we would require a total sample of 700 patients. Assuming a 10% loss to follow-up rate, we required a total sample of 800 patients (400 EPL cases and 400 NEPL cases).

Descriptive statistics were used, and data was presented as n (%) and mean ± standard error of the mean (SEM) as indicated. Maternal variables, stratified by primary language, were compared using Student’s t-test and Mann–Whitney U test for continuous variables, and Chi-square and Fisher’s exact test for categorical variables, as indicated. Multivariable logistic regression was performed in order to assess the primary and secondary outcomes as well as examine their association with maternal language after adjusting for confounders. Associations are shown as adjusted odds ratios (aOR) and 95% confidence intervals. Two-sided P values of < 0.05 were considered statistically significant. SPSS (Statistical Package for the Social Sciences) (IBM SPSS Statistics for Macintosh, version 29.0; IBM Corp, Armonk, NY) was used for statistical analysis. We used the Strengthening The Reporting of Observational Studies in Epidemiology (STROBE) guidelines in our study design and documentation (Von Elm et al., 2007).

## Results

A total of 1723 patients with PPH between January 2020 and December 2022 met inclusion criteria: 1314 (76.3%) had EPL and 409 (23.7%) had NEPL. Among those with NEPL, 332 (80.1%) were primary Spanish-speaking, followed by 26 (6.4%) Bengali, 25 (6.1%) Arabic, 15 (3.7%) French and 11 (2.7%) speaking other languages.

Table 1 summarizes demographic, maternal, and obstetric characteristics between the two groups. There were no differences between groups in age, parity, gestational age at delivery, mode of delivery, and incidence of preterm delivery or multiple gestation. In addition, incidence of diabetes, chronic hypertension, placenta abruption and chorioamnionitis were similar between language groups. Patients with NEPL were more likely to be of Hispanic race (75.1% vs 41.6%, p < 0.001), and had a lower body mass index (BMI) (33.2 ± 6.2 vs 35.3 ± 7.6, p < 0.001) and incidence of preeclampsia (16.9% vs 24.5%, p = 0.001), when compared with English–speaking patients. Etiology of PPH was similar between non-English and English primary language groups, with uterine atony as the most common cause (55.7% vs 52.4%, p = 0.2). Total postpartum blood loss was similar between groups (1496.0 ± 668.1 vs 1530.2 ± 634.2, p = 0.3).

**Table 1:** Demographic, maternal and gestational characteristics amongst patients with postpartum hemorrhage stratified by English and non-English primary language

Characteristic	English primary language (n = 1314)	Non-English primary language (n = 409)	P value
Age, years (mean ± SEM)	31.7 ± 6.3	31.6 ± 6.0	0.6
Parity (mean ± SEM)	1.2 ± 1.3	1.3 ± 1.2	0.08
Race/Ethnicity			
Non-Hispanic white	63 (4.8)	8 (2.0)	< 0.001
Non-Hispanic Black	502 (38.2)	14 (3.4)	
Hispanic	547 (41.6)	307 (75.1)	
Asian	47 (3.6)	50 (12.2)	
Other	155 (11.8)	30 (7.3)	
BMI, kg/m^2^ (mean ± SEM)	35.2504 ± 7.58327	33.1809 ± 6.21353	< 0.001
Gestational age at delivery, weeks (mean ± SEM)	38.0 ± 2.6	38.0 ± 2.8	0.9
Preterm delivery	211 (16.1)	57 (14.0)	0.3
Twin gestation	74 (5.6)	18 (4.4)	0.3
Mode of delivery			
Vaginal	178 (13.6)	56 (13.7)	0.9
Cesarean section	1136 (86.5)	353 (86.3)	
Pre-delivery hemoglobin	11.6 ± 0.3	11.8 ± 0.5	0.2
Diabetes	303 (23.1)	111 (27.1)	0.1
Chronic hypertension	180 (13.7)	41 (10.0)	0.1
Preeclampsia	322 (24.5)	69 (16.9)	0.001
Placental abruption	51 (3.9)	11 (2.7)	0.3
Chorioamnionitis	186 (14.2)	47 (11.5)	0.2
Quantitative blood loss	1530.2 ± 634.2	1496.0 ± 668.1	0.3

*BMI* body mass index

Data presented as n (%), unless otherwise specified

Maternal adverse outcomes, stratified by primary language, are detailed in Table 2. Non English–speaking patients with PPH were less likely to receive a postpartum blood transfusion (23.0% 28.2%, p = 0.039), when compared with English-speaking patients with PPH. 38 (3.0%) patients in the English-speaking group and 8 (2.0%) patients in the non-English-speaking group declined blood transfusion, despite provider recommendation. Incidence of blood transfusion of 4 or more units, DIC and ICU admission were similar between language groups.

**Table 2:** Rate of markers of severe maternal morbidity amongst patients with postpartum hemorrhage, stratified by English and non-English primary language

Characteristic	English primary language (n = 1314)	Non-English primary language (n = 409)	P value
Blood transfusion	370 (28.2)	94 (23.0)	0.04
Massive transfusion (≥ 4 units)	36 (2.7)	7 (1.7)	0.2
Disseminated intravascular coagulation	15 (1.1)	6 (1.5)	0.6
ICU admission	14 (1.1)	5 (1.2)	0.8

*ICU* intensive care unit

Data presented as n (%)

Table 3 presents the results of our multivariable logistic regression analysis on the risk of severe maternal morbidity outcomes in patients with PPH, stratified by primary language. After controlling for age, race/ethnicity, nulliparity, BMI, pre-delivery anemia and preeclampsia, NEPL was associated with decreased odds of blood transfusion in PPH, when compared to patients with EPL. Adjusted analyses did not show a difference between language groups in odds of blood transfusion of 4 or more units, DIC or admission to the ICU.

**Table 3:** Multivariable logistic regression analysis of the risk of severe maternal morbidity in patients with postpartum hemorrhage, stratified by English and non-English primary language

	English primary language	Non-English primary language					
		Odds ratio (OR)	95% CI	p-value	Adjusted odds ratio (aOR)	95% CI	P value
Blood transfusion	Ref	0.8	0.60–0.98	0.04	0.7	0.55–0.98	0.04
Massive transfusion (≥ 4 units)	Ref	0.6	0.27–1.40	0.2	0.7	0.28–1.80	0.5
Disseminated intravascular coagulation	Ref	1.3	0.50–3.35	0.6	1.4	0.47–4.44	0.5
ICU admission	Ref	1.1	0.41–3.21	0.8	1.2	0.36–3.77	0.8

*ICU* intensive care unit

Data presented as n (%)

## Discussion

In our study we found that amongst patients experiencing PPH, despite a similar postpartum blood loss, patients with a non-English primary language were less likely to receive a blood transfusion compared to those with an English primary language. However, despite this difference in blood transfusion administration, other markers of severe maternal morbidity (massive transfusion, DIC and ICU admission) did not differ by primary language preference.

There are several explanations for the observed variation by primary language in transfusion practices with PPH despite similar blood loss. Hospital factors may include a lack of language-concordant providers and subsequent difficulty with effective communication, clinical bias, and differential access to phone or in-person interpreter services (Comfort et al., 2023; Schaefer et al., 2021). Moreover, individual patient factors like language proficiency and literacy, wavering cultural norms and co-morbidities (like obesity, anemia and preeclampsia, all of which were controlled for in our analysis), may contribute as well. Finally, religious beliefs and cultural preferences may also contribute to declining blood transfusion, even in the setting of hemorrhage. In our study, the percentage of patients declining a transfusion was similar among English and non-English-speaking patients.

Prior studies in obstetrics have suggested language barriers contribute to care inequities. Sentell et al. (2015) demonstrated increased obstetric birth trauma among non-English-speaking Asian and Pacific Islander patients in Hawaii, while Eslier et al. (2023) found decreased prenatal care utilization amongst non-French-speaking migrant patients in Paris. Lastly, Comfort et al. (2023) showed increased unscheduled cesarean delivery amongst non-English-speaking nulliparous patients in the Bronx. Similar to these studies, in our racially and ethnically diverse patient population, non-English-speaking patients whose birth was complicated by hemorrhage were less likely to receive necessary treatment with blood transfusion compared to their English-speaking counterparts, despite a similar blood loss.

Understanding sociodemographic factors that contribute to healthcare inequities is imperative to improve care for marginalized groups. Racial and ethnic disparities resulting in decreased care escalation and transfusion with PPH have been documented in the literature (Guan et al., 2023; Miller et al., 2022), highlighting that structural racism, discrimination, and implicit bias directly correlate with poor patient outcomes. Our results shine a light on an additional sociodemographic variable, primary language, which may further contribute to care inequities. Racially and ethnically diverse clinicians, as well as the implementation of verified phone or in-person interpreters that communicate in the patient’s preferred language, may eliminate inequities in the treatment of patients whose birth is complicated by obstetric hemorrhage.

Our study is novel in examining the aspect of language and morbidity in obstetric hemorrhage. Strengths of our study include a large and ethnically diverse patient population in an urban setting. Our sample size is well-powered for the primary outcome of blood transfusion in PPH. To decrease individual bias, our PPH cohort was obtained using the American College of Obstetrics and Gynecology’s (ACOG) objective criteria of QBL of greater than 1000 ml of blood instead of the provider estimation of blood loss. Our study population included patients after ACOG’s updated bulletin in 2017 recommending standardized obstetric care bundles in PPH to decrease disparities (Postpartum Hemorrhage, 2017).

Despite a well-developed study, there are also limitations. Our study is retrospective, which allows for potential unintended selection and misclassification bias. Primary language information was obtained via chart review from the EMR, and we understand there is potential for incorrect documentation and language classification. In addition, we were only able to assess language preference and not language proficiency; therefore it is possible that patients with a documented English primary language were unable to communicate effectively in English. We did not have data on provider language preference as within our academic system patients potentially interact with multiple attending and house-staff physicians, nurses, and medical assistants throughout hospitalization. We acknowledge there is overlap between different sociodemographic factors including race and/or ethnicity, which we attempted to control for in our analysis. Lastly, originating from only one urban academic hospital system may limit the scope and generalizability of our findings to other healthcare models.

In our study, we found that amongst patients with PPH, despite a similar blood loss, non-English-speaking patients were less likely to receive a blood transfusion, compared to English speakers. Further prospective studies are needed to explore language and communication disparities in care amongst patients with PPH within the larger population.

## Data Availability

The datasets used and/or analyzed during the current study are available from the corresponding author on reasonable request.

## Abbreviations

PPH: Postpartum hemorrhage
DIC: Disseminated intravascular coagulation
ICU: Intensive care unit
EMR: Electronic medical record
QBL: Quantitative blood loss
EPL: English primary language
NEPL: Non-English primary language
pRBCS: Packed red blood cells
SEM: Standard error of the mean
STROBE: Strengthening The Reporting of Observational studies in Epidemiology
ACOG: American College of Obstetrics & Gynecology
